# Functional Comparison of Human and Zebra Fish FKBP52 Confirms the Importance of the Proline-Rich Loop for Regulation of Steroid Hormone Receptor Activity

**DOI:** 10.3390/ijms20215346

**Published:** 2019-10-28

**Authors:** Diondra C. Harris, Yenni A. Garcia, Cheryl Storer Samaniego, Veronica W. Rowlett, Nina R. Ortiz, Ashley N. Payan, Tatsuya Maehigashi, Marc B. Cox

**Affiliations:** 1 Border Biomedical Research Center and Department of Biological Sciences, University of Texas at El Paso, El Paso, TX 79968, USA; diondra.harris@gmail.com (D.C.H.); yagarcia@chem.ucla.edu (Y.A.G.); csamaniego@kettering.edu (C.S.S.); veronicarowlett@gmail.com (V.W.R.); nrortiz@miners.utep.edu (N.R.O.); anpayan@miners.utep.edu (A.N.P.); 2 Department of Chemistry and Biochemistry, Kettering University, Flint, MI 48504, USA; 3 Department of Pediatrics, School of Medicine, Emory University, Atlanta, GA 30322, USA; t.maehigashi@gmail.com

**Keywords:** Danio rerio, zebra fish, steroid hormone receptor, cochaperone, FKBP52, heat shock protein 90 (Hsp90), Hsp70, proline-rich loop, androgen receptor, glucocorticoid receptor, progesterone receptor

## Abstract

Previous studies demonstrated that the 52-kDa FK506-binding protein (FKBP52) proline-rich loop is functionally relevant in the regulation of steroid hormone receptor activity. While zebra fish (*Danio rerio; Dr*) FKBP52 contains all of the analogous domains and residues previously identified as critical for FKBP52 potentiation of receptor activity, it fails to potentiate activity. Thus, we used a cross-species comparative approach to assess the residues that are functionally critical for FKBP52 function. Random selection of gain-of-function DrFKBP52 mutants in *Saccharomyces cerevisiae* identified two critical residues, alanine 111 (A111) and threonine 157 (T157), for activation of receptor potentiation by DrFKBP52. In silico homology modeling suggests that alanine to valine substitution at position 111 in DrFKBP52 induces an open conformation of the proline-rich loop surface similar to that observed on human FKBP52, which may allow for sufficient surface area and increased hydrophobicity for interactions within the receptor–chaperone complex. A second mutation in the FKBP12-like domain 2 (FK2), threonine 157 to arginine (T157R), also enhanced potentiation, and the DrFKBP52-A111V/T157R double mutant potentiated receptor activity similar to human FKBP52. Collectively, these results confirm the functional importance of the FKBP52 proline-rich loop, suggest that an open conformation on the proline-rich loop surface is a predictor of activity, and highlight the importance of an additional residue within the FK2 domain.

## 1. Introduction

Steroid hormone receptors are ligand-dependent transcription factors that require the dynamic and highly ordered assembly of multiple chaperone and cochaperone heterocomplexes to reach a functional conformation [1]. At least twelve proteins in at least three distinct complexes contribute to receptor folding and/or holding within the cytoplasm. The mature complex in which the receptor is capable of high-affinity hormone-binding includes the 90-kDa heat shock protein (Hsp90), the Hsp90-associated 23-kDA cochaperone (p23), and one of a family of tetratricopeptide repeat (TPR)-containing proteins termed immunophilins. Unlike most of the Hsp90-associated cochaperones, the TPR-containing cochaperones that present in the mature receptor-Hsp90 complex are functionally specific for a subset of Hsp90 client proteins [2]. As a result, Hsp90 is thought to sample the available pool of TPR-containing cochaperones, and the client protein present in the complex determines which TPR-containing cochaperone will functionally interact.

The 52-kDa FK506-binding protein (FKBP52) associates with receptor-Hsp90 complexes by way of a C-terminal TPR domain [3,4] and is a specific positive regulator of androgen receptor (AR), glucocorticoid receptor (GR), and progesterone receptor (PR) signaling in cellular assays [5,6,7]. These results have been confirmed in animals as *fkbp52*-deficient (52KO) mice display phenotypes related to reduced AR, PR, and GR signaling [6,8]. The specific mechanism by which FKBP52 functionally influences receptor activity is unknown, but it is likely that FKBP52 participates in multiple distinct steps within the receptor signaling pathways. Data suggest that FKBP52 not only influences receptor hormone-binding affinity within the mature Hsp90 complex but also participates in hormone-induced receptor translocation to the nucleus through direct interactions with the dynein motor protein [9,10]. In addition, FKBP52 was recently demonstrated to interact directly with β-catenin to promote β-catenin interaction with, and upregulation of AR activity [11]. Interestingly, this mechanism was independent of FKBP52 binding to Hsp90. Despite the multiple roles identified for FKBP52, it is clear that FKBP52 is a functionally critical factor for AR, GR, and PR activity at physiological hormone concentrations in vivo.

Studies with chimeric receptor proteins demonstrated that FKBP52-mediated effects localized to the receptor hormone-binding domain [5]. In support of this idea, we previously demonstrated that mutations on the AR binding function 3 (BF3) surface cause increased dependence (also termed FKBP52 hypersensitivity) on FKBP52 for function [6,12]. We also identified and characterized a series of small molecules that specifically inhibit FKBP52-mediated potentiation of AR signaling, and available evidence suggests that these molecules target the AR BF3 surface [12]. Taken together, these findings suggest that the AR BF3 surface is an FKBP52 interaction and/or regulatory surface. The Bag-1L protein was also recently found to regulate AR activity through the BF3 surface, suggesting that the AR BF3 surface may serve as a promiscuous regulatory surface for several factors, including FKBP52 [13]. Mutations within the H1–H3 loop in the GR hormone-binding domain were also shown to influence FKBP52-mediated receptor activity [14]. The H1–H3 loop may act as a regulatory surface that promotes allosteric conformational changes in BF3, given that FKBP52 does not directly interact at the H1–H3 loop. However, evidence suggests that the co-regulation of AR by FKBP52 and β-catenin through the AR BF3 surface is a specific AR regulatory mechanism [11,15]. Thus, FKBP52 may influence the activity of multiple receptors through multiple distinct sites, including the H1–H3 loop.

A detailed understanding of the residues and domains that contribute to the FKBP52 function can inform ongoing efforts to therapeutically target FKBP52 for the disruption of receptor activity. FKBP52 shares approximately 70% sequence similarity to FKBP51, yet FKBP51 overexpression does not potentiate receptor function in our yeast and mammalian cell assay systems. Thus, this functional difference was exploited in previous studies to map the domains and residues on FKBP52 that are required for function [5,16]. The FKBP52 N-terminal FK506 binding domain (FK1) is required for receptor regulation, and functional domain mapping studies demonstrated that two mutations (A116V and L119P), combined were sufficient to confer full receptor potentiating activity to FKBP51. The analogous residues on FKBP52 are within a proline-rich loop (also termed 80 s loop or β4–β5 loop) overhanging the peptidyl-prolyl *cis*/*trans* isomerase (PPIase) catalytic pocket, suggesting that the proline-rich loop is critical for function. PPIase enzymatic activity is not required for function, but the FKBP ligand and PPIase inhibitor FK506 disrupts receptor potentiation, possibly through the disruption of FK1 and/or proline-rich loop interactions within receptor complexes. Although this region was identified as important for FKBP52 function, the converse mutations in FKBP52 only reduced potentiation by approximately 40%. Thus, the FK1 proline-rich loop is necessary for normal FKBP52 function, but it is not sufficient alone to generate a full response. Thus, other residues likely exist within the FKBP52 FK1 and/or FK2 domains that are required for full FKBP52 function. This is further supported by the lack of receptor potentiating activity of an FKBP52 ortholog from zebra fish.

Zebra fish (*Danio rerio*; Dr) FKBP52 is identical to human (*Homo sapiens*; Hs) FKBP52 with respect to all known residues that are critical for receptor potentiation. However, as detailed here, DrFKBP52 fails to potentiate receptor activity in reporter assays. Taking advantage of this functional difference, the genetic selection of gain-of-function random DrFKBP52 mutants was used to identify and characterize additional residues and/or domains that are critical for FKBP52 potentiation of receptor activity.

## 2. Results

### 2.1. Danio Rerio FKBP52: a Comparative Model for Mapping Human FKBP52 Functional Residues/Domains

**Figure 1:**
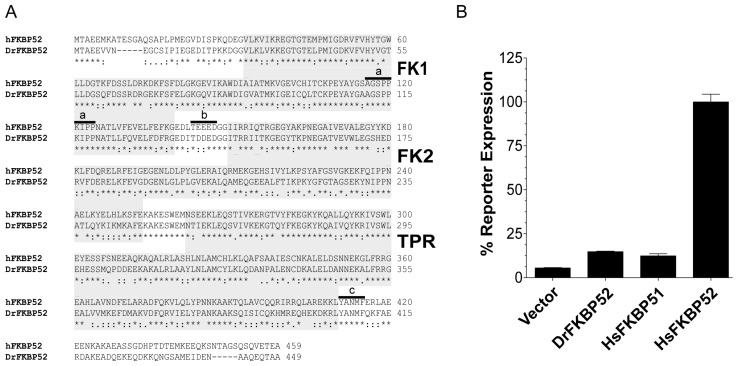
DrFKBP52 contains all known critical domains and residues for function but does not potentiate receptor activity. (**A**) Amino acid sequence alignment for human FKBP52 (HsFKBP52, Genbank accession number NP_002005.1) and *Danio rerio* FKBP52 (DrFKBP52, Genbank accession number NP_958877) indicates that DrFKBP52 has 61% similarity to HsFKBP52. The FK1, FK2, and TPR domains are indicated in gray. Previously characterized domains critical for function including the proline-rich loop (a), FK linker and CKII phosphorylation site (b), and conserved C-terminal tail motif important for Hsp90 binding (c) are indicated. (**B**) Dihydrotestosterone-induced AR-mediated β-galactosidase reporter assays were performed in yeast in the presence or absence of the indicated expression vectors for DrFKBP52, HsFKBP52, and HsFKBP51. In all cases, androgen receptor signaling in cells expressing HsFKBP52 was significantly higher (*p* ≤ 0.0001) as compared with cells expressing Vector, HsFKBP51, and DrFKBP52.

Previous FKBP51 gain-of-function random mutagenesis studies demonstrated the importance of the FKBP52 proline-rich loop in the regulation of steroid hormone receptor activity [16]. However, chimeric studies with FKBP51 and FKBP52 suggested that additional functionally required residues may exist within the FKBP52 FK2 domain. Thus, we used multiple sequence alignments and functional assays to identify additional FKBP52 orthologs that could be used for similar comparative studies. DrFKBP52 shares approximately 61% similarity in amino acid identity to HsFKBP52. In addition, DrFKBP52 contains all of the regions and residues previously identified as critical for HsFKBP52 function including the six amino acids that comprise the proline-rich loop, a consensus casein kinase II (CKII) phosphorylation site [17], an 85% similar FK2 domain that lacks PPIase catalytic activity, and the C-terminal YANMF motif that binds Hsp90 [4] (Figure 1A). Despite their similarity at the amino acid level, DrFKBP52 and HsFKBP52 are functionally distinct as, unlike HsFKBP52, DrFKBP52 does not potentiate human AR activity in yeast-based AR-mediated reporter assays (Figure 1B). This functional distinction can be exploited for functional residue and domain mapping studies.

### 2.2. Random Selection of Gain-of-Function DrFKBP52 Mutants

**Figure 2:**
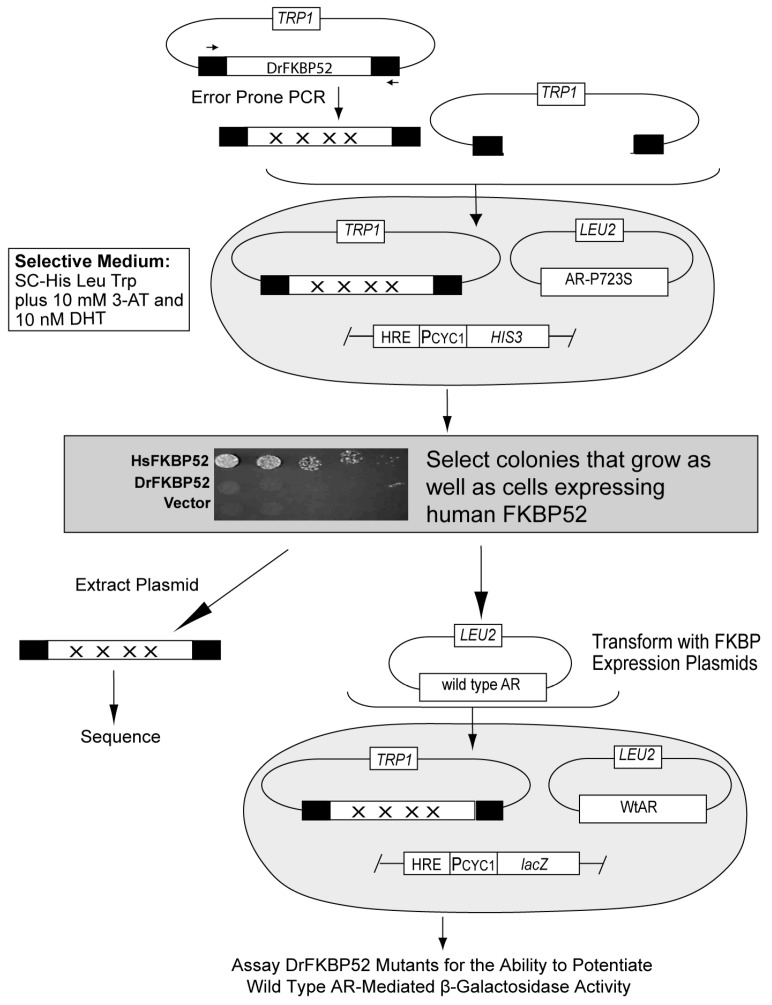
Selection scheme for *Danio rerio* FKBP52 gain-of-function mutants. Libraries of random *Dr*FKBP52 mutants were independently generated by error-prone PCR using primers (horizontal arrows) binding upstream in the GAPDH promoter (PGPD) or downstream in the transcriptional terminator (Term). Randomly generated mutant constructs were co-transformed with a linearized vector to facilitate homologous recombination between the common promoter and terminator regions on these fragments reconstituting *TRP1*-marked expression plasmids harboring the DrFKBP52 mutants. The parental strain contains a *LEU2*-marked androgen receptor (AR)-P723S expression plasmid and integrated *HIS3* reporter gene driven by a hormone-responsive promoter element (HRE) such that growth in histidine-lacking medium is dependent on AR-P723S activity. Transformants were plated on selective growth medium supplemented with 10 mM 3-amino-1,2,4-triazole and 10 nM dihydrotestosterone (DHT), and colonies that grew, as well as those expressing human FKBP52, were selected for further analysis. Mutants exhibiting the gain-of-function phenotype were extracted from yeast and co-transformed with wild type AR into a secondary strain containing a hormone-responsive LacZ reporter plasmid, and assayed for the ability to potentiate wild type AR-mediated β-galactosidase activity. DrFKBP52 mutants that gained the ability to potentiate AR activity in these assays were sequenced to identify relevant mutations (inset). Yeast strains containing a hormone-inducible *HIS3* gene and expressing AR-P723S plus either Vector, HsFKBP52, or DrFKBP52 were serially diluted and spotted on selective medium containing a growth-limiting concentration of 10 nM DHT (inset).

Taking advantage of the functional divergence between DrFKBP52 and HsFKBP52, we used yeast genetics to identify residues functionally critical for potentiation of receptor activity by selecting for gain-of-function mutations in DrFKBP52 that conferred the ability to potentiate receptor activity similar to HsFKBP52. The selection strategy used was modified from the strategy previously used to select for gain-of-function FKBP51 mutations [16] and is illustrated in Figure 2. The strain used contains both a stable AR-mediated *HIS3* gene for use in selecting for dihydrotestosterone (DHT)-dependent growth in histidine-lacking medium (selection of gain-of-function mutations) and a stable AR-mediated β-galactosidase reporter gene for independently verifying and quantifying the DrFKBP52 mutant phenotypes. To enhance the sensitivity of the selection strategy, 3-AT was added to the medium to inhibit growth due to leaky *HIS3* gene expression. In addition, the selection strain expressed AR-P723S, which is hyper-dependent on FKBP52 for function and increases the sensitivity of the selection [6,12,16]. Thus, in the presence of 10 nM DHT, only growth from those cells expressing HsFKBP52, which potentiates DHT-dependent, AR-mediated *HIS3* expression was detectable on histidine-lacking medium, while DrFKBP52 supported very little to no growth under these same conditions (Figure 2; inset).

To generate DrFKBP52 random mutants that could support DHT-dependent growth on histidine-lacking medium, we used error-prone PCR under conditions optimized to produce three to five mutations per PCR product. The DrFKBP52 mutant library was transformed into the selection strain and plated on selective medium. Those colonies that appeared under the indicated selective conditions were cloned, and the DrFKBP52 expression plasmid was extracted and sequenced to identify the random mutations present in each clone. To verify and quantify the gain-of-function phenotype, and to ensure that DrFKBP52 mutant potentiation is not yeast strain-dependent, each DrFKBP52 mutant expression plasmid was co-transformed into a clean genetic background with a wild type AR expression plasmid and assayed for DHT-dependent β-galactosidase reporter gene expression.

**Figure 3:**
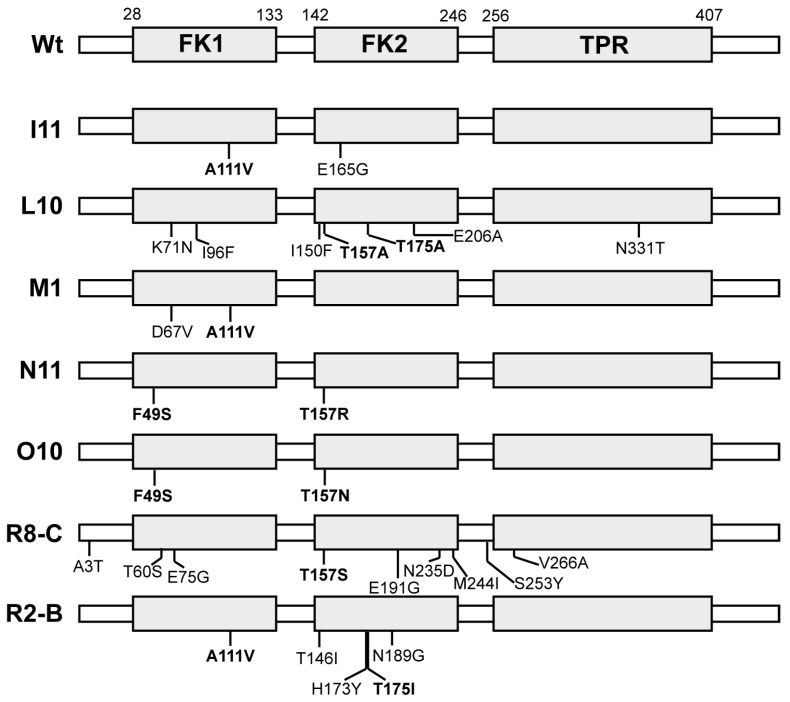
DrFKBP52 gain-of-function mutants isolated. The domain arrangement of wild type DrFKBP52 with amino acid numbering at the domain boundaries is illustrated at the top with identified mutants aligned below. Each mutant is coded based on the independent library from which it was isolated (I to R) and an isolate number. Repeatedly identified mutations of particular interest are shown in bold.

Gain-of-function DrFKBP52 mutants were isolated from numerous independent error-prone PCR reactions, with the expectation that key mutations would be commonly identified across multiple, independent PCR reactions. A total of 34 gain-of-function mutants were isolated from ten independent libraries; of these, five had common residues mutated in FK1 (F49S and A116V/T), and five had common residues mutated in FK2 (T157A/R and T175A/I) (Figure 3). The DrFKBP52 A111 residue is analogous to A116 in HsFKBP52 and HsFKBP51. Interestingly, mutation of this residue to valine (A116V) was previously found to confer full receptor potentiating ability to FKBP51 when combined with the L119P mutation. The DrFKBP52 T157 residue is analogous to A162 in HsFKBP52 and S160 in HsFKBP51. While nothing has been reported previously regarding this residue, this is a conserved residue across all three proteins.

### 2.3. Characterization of DrFKBP52 Mutants

**Figure 4:**
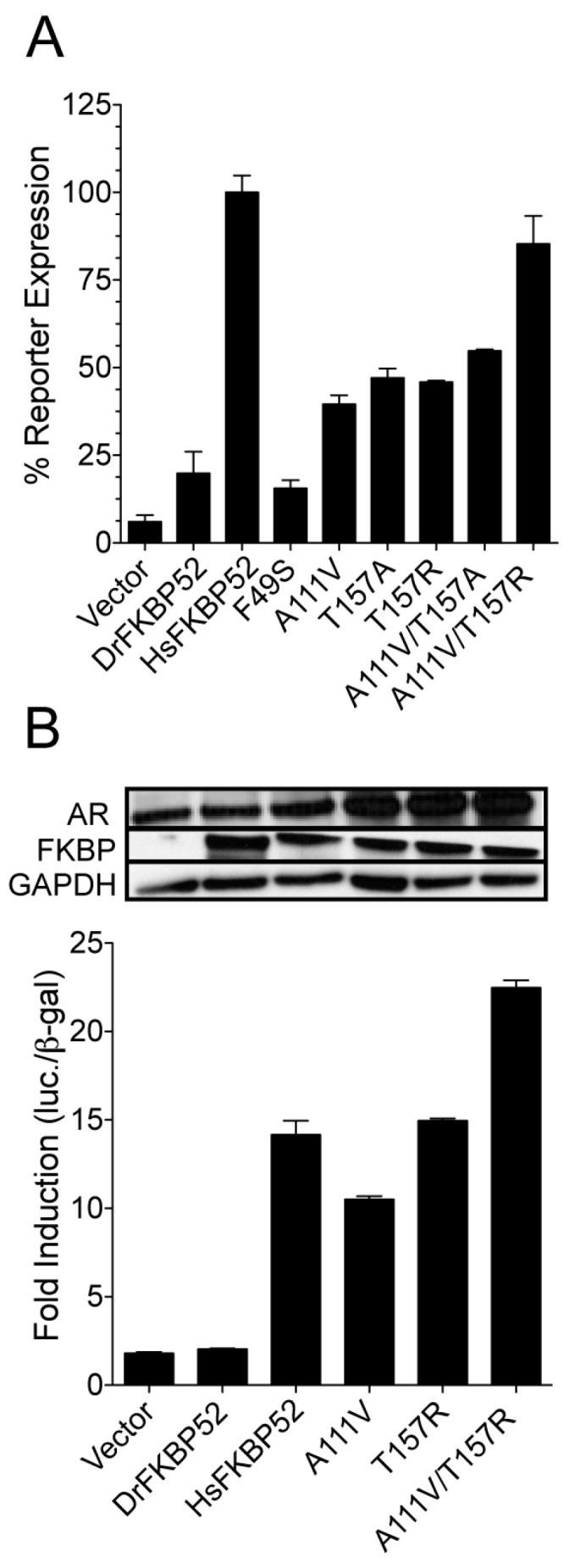
DrFKBP52 mutants potentiate androgen receptor activity. (**A**) Hormone-dependent β-galactosidase reporter gene activity was measured in yeast strains expressing wild type AR in the presence of 3 nM DHT. (**B**) FKBP activities were similarly determined in transfected 52KO MEF cells expressing wild-type AR and a hormone-dependent luciferase reporter. Protein expression levels were monitored by Western blot for the introduced FKBPs, AR, and endogenous GAPDH as a loading control. In all cases (A and B), androgen receptor activity in cells expressing DrFKBP52-A111V/T157R was significantly higher (*p* ≤ 0.0001) as compared with cells expressing Vector and DrFKBP52. There was no significant difference in androgen receptor activity in cells expressing human FKBP52 and DrFKBP52-A111V/T157R.

Given that the selection of gain-of-function mutants was designed to identify constructs with multiple mutations, we next generated the individual mutations by site-directed mutagenesis to confirm the individual role of each identified mutation in potentiating wild type human AR activity. Thus, each individual mutation was assessed for the ability to potentiate wild type AR activity in yeast-based, AR-mediated β-galactosidase reporter assays (Figure 4A). DrFKBP52-A111V and T157R individually potentiated AR signaling three- to five-fold higher than that of wild type DrFKBP52. The DrFKBP52-A111V/T157R double mutant potentiated AR activity to levels comparable to that of HsFKBP52, thereby restoring full receptor potentiating ability in yeast. While DrFKBP52-T157A and T157R individually supported similar levels of AR activity, only DrFKBP52-T157R displayed additive effects when combined with the A111V mutation. A number of false positives were also identified in the screen shown in Figure 2. For example, the DrFKBP52-F49S mutation had no effect on wild type AR activity in these assays (Figure 4A). The mutations at position T175 highlighted in Figure 3 also displayed no activity. Given that the AR-P723S mutant was used in the screen, it is likely that these mutants specifically affected AR-P723S, but not the more physiologically relevant wild type AR. Thus, no further analysis was performed on these mutants.

To corroborate these findings in a higher vertebrate model system, a similar analysis was performed in *fkbp52*-deficient mouse embryonic fibroblasts (52KO MEF) in which DrFKBP52A111V and T157R were able to individually potentiate AR-mediated luciferase activity, and, when combined, potentiated activity comparable to HsFKBP52 (Figure 4B). Western blot analysis indicated that the gain-of-function observed with the DrFKBP52 mutants is not likely due to differences in FKBP52 and AR protein stability.

### 2.4. Homology Modeling of FKBPs

**Figure 5:**
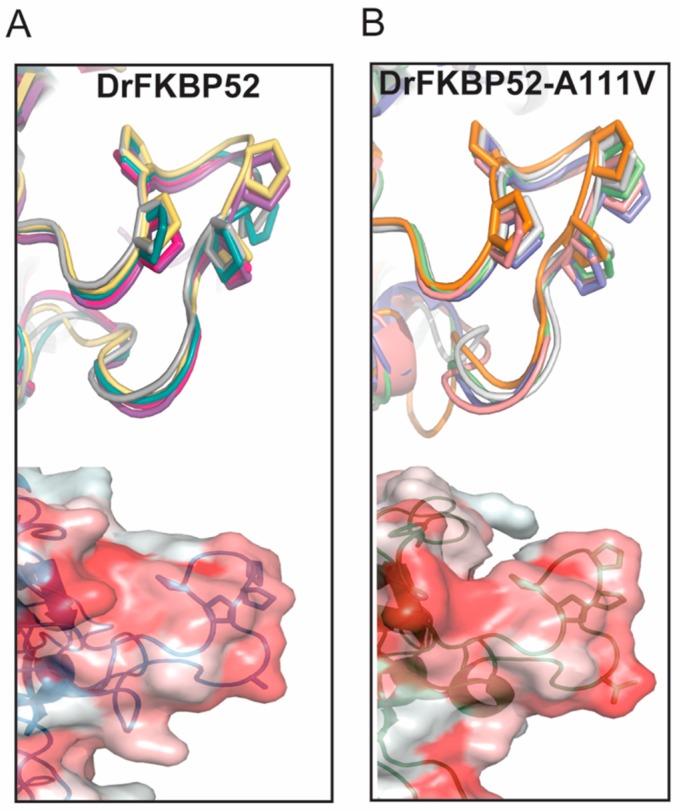
Predictive modeling and hydrophobicity scale of DrFKBP52 and DrFKBP52-A111V. The top structures represent the top five 3D models for each structural query of DrFKBP52 (**A**) and DrFKBP52-A111V (**B**) predicted by I-TASSER. The hydrophobicity on the surface of the loop was assessed (red = hydrophobic) in DrFKBP52 (**A**) and DrFKBP52-A111V (**B**) to allow us to further evaluate the structural changes that may be relevant in their functional divergence (bottom structures).

Structural studies have confirmed differences between FKBP51 and FKBP52 at the B4–B5 loop (Proline-rich loop). Proline 119, and Proline 120 were reported to be major determinants of functional opposing effects between FKBP51 and FKBP52 [18,19]. However, only A116V and L119P mutations resulted in HsFKBP51 full activity in functional assays [16]. The significance of alanine to valine at position 116 remains unknown. Thus, we used homology modeling to generate the structure of DrFKBP52 and DrFKBP52-A111V and compared the predicted proline-rich loop structures to those of HsFKBP51 and HsFKBP52 to further understand the structural significance of residue A111/A116 in the FKBPs. I-TASSER predicted five 3D models for each structural query, DrFKBP52 and DrFKBP52-A111V (Figure 5). The loop region was stacked to determine whether the loop region maintains a similar conformation in all predictive models. While slight variations were seen, they are insignificant, indicating that homology modeling for each model kept a similar conformation.

**Figure 6:**
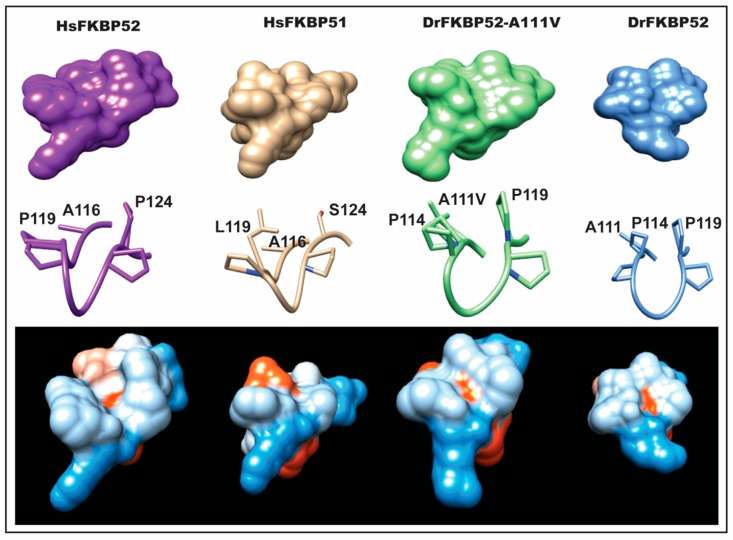
Predicted structural differences in the FK1 proline-rich loop. Homology modeling was used to generate predicted models of DrFKBP52 and DrFKBP52-A111V, in the comparison to human FKBP52 and FKBP51 to determine conformational changes induced by the DrFKBP52-A111V mutation. Crystal structures of HsFKBP51 (PDB ID: 1KT0), HsFKBP52 (PDB ID: 1Q1C), DrFKBP52 (predicted), and DrFKBP52-A111V (predicted) are aligned; the respective FK1 domains are shown in space-filled modeling and colored in hydrophobicity scale (blue = hydrophilic, red = hydrophobic). Note that the only differences between FKBP51 and HsFKBP52 within the loop region are at positions 119 and 124.

Because A111V mutation is located at the proline-rich loop region, only the loop was isolated for further analysis (Figure 6). The valine substitution at position 111 is predicted to affect the surface charge (to more neutral) and the hydrophobicity (to more hydrophobic) in the vicinity. The top space-filled models showed a closed conformation for both HsFKBP51 and DrFKBP52, while HsFKBP52 had a more open conformation. The same is seen in the stick model; HsFKBP51 L119 and S124 were in proximity above the loop region, and analogous residues in DrFKBP52 (P114 and P119) were also in proximity above the loop. However, the DrFKBP52-A111V mutation was predicted to induce a distinct conformational change. This mutant takes on an open conformation similar to that of HsFKBP52; both the space-filled and stick models showed an unobstructed pocket above the proline-loop. This was further corroborated by hydrophobic surface calculation, which predicted that both the HsFKBP52 and DrFKBP52-A111V, but not DrFKBP52 or HsFKBP51, proline-loop forms a hydrophobic pocket, possibly facilitating interaction at this surface.

## 3. Discussion

Human FKBP52 is an Hsp90 cochaperone that positively regulates hormone-dependent transcriptional activity of AR, GR, and PR. Its physiological relevance has been established in studies showing impaired reproductive development of male and female *fkbp52*-deficient mice [6,7,8,20,21]. While the specific mechanisms for receptor potentiation are still poorly understood, studies implicate FKBP52 in the regulation of receptor–ligand binding and trafficking (reviewed in [10]). FKBP52 assembles with receptor complexes through its association with Hsp90 to exert effects on receptor activity. In particular, the N-terminal FK1 PPIase domain of FKBP52 has been recognized as important for activity [16]. While the PPIase enzymatic activity was not required for FKBP52 regulation of receptor activity, these studies highlighted the importance of the proline-rich loop within the FK1 domain. Introducing both the A116V and L119P mutations in the FKBP51 proline-rich loop conferred full receptor potentiating ability similar to that seen with wild type FKBP52. While the L119P mutation made FKBP51 more like FKBP52 at this site, the significance of the A116V mutation was still unclear given that FKBP52 also has an alanine at this position. In addition to the proline-rich loop, studies suggested that the short FK-linker between the FK1 and FK2 domains behaves as a flexible hinge region that may facilitate non-covalent interactions between the FK1 and FK2 domains, revealing a potential role for the FK2 domain in facilitating a functional FK1 conformation [5,22,23].

To further elucidate the domain and residue requirements for FKBP52 potentiation of receptor activity, we took a cross-species comparative approach. Despite the presence of the proline-rich loop, FK linker, Hsp90 binding motif, and predicted structural similarities to HsFKBP52, DrFKBP52 still does not potentiate receptor activity (Figure 1). Taking advantage of this functional distinction, DrFKBP52 was randomly mutated, and a yeast selection screen was designed to isolate DrFKBP52 mutants that gain receptor potentiation activity (Figure 2). The gain-of-function mutants that were repeatedly identified were A111V (nine times) and T157R (seven times) (Figure 3). Separately these mutants had sufficient activity to increase potentiation of AR activity three- to five-fold as compared to wild type DrFKBP52 (Figure 4). Interestingly, the DrFKBP52-A111V/T157R double mutant completely restored activity to levels similar to HsFKBP52 in both yeast and mammalian cells. It should be noted that while the T157A mutant was able to confer receptor potentiation activity separately, combining this mutation with A111V did not restore full activity as compared to HsFKBP52. Given the flexibility of the hinge region, these data suggest a possible salt bridge between the FK1 and FK2 domains may specifically affect FKBP52 regulation of steroid hormone receptor activity. Interestingly, T157 is located down-stream of IRRIQTR, a highly positively charged region that, like the proline-rich loop region, is highly conserved in FKBP52 and FKBP51 across species, though some are more positively charged in this region. Though we have not confirmed a specific residue in the FK2 domain that is important for HsFKBP52 potentiation, we postulate that residues near the FK-linker region may facilitate FK1-FK2 interactions that allosterically affect FK1 proline-rich loop conformation, as previous studies suggest [18]. The T157R mutation in Dr-FKBP52 could be enhancing such an interaction between FK1 and FK2.

Interestingly, A111 in DrFKBP52 is analogous to A116 in HsFKBP51 and HsFKBP52, a proline-rich loop residue previously highlighted by Riggs et al. [16]. Because wild type DrFKBP52, HsFKBP51, and HsFKBP52 all contain alanine at loop position 111/116, we performed homology modeling to predict the role of this amino acid position may play in determining the proline-rich loop structure (Figure 5 and Figure 6). A comparison of the corresponding loops in HsFKBP52, HsFKBP51, DrFKBP52, and DrFKBP52-A111V revealed a predicted structural difference due to the respective amino acids at position 111/116. The DrFKBP52 proline side chains (P114 and P119) protrude into a hydrophobic notch that forms along the top of the loop, more similar to HsFKBP51. The projection of these two residues is significantly altered by the addition of valine at position 111 in DrFKBP52. The conformation of the analogous prolines in DrFKBP52-A111V spread more outward, similar to HsFKBP52. Hydrophobic surface depictions show that HsFKBP52, HsFKBP51, DrFKBP52, and DrFKBP52-A111V all possess a hydrophobic notch on the surface of the proline loop. This surface is blocked by the projection of surrounding residues in DrFKBP52 and HsFKBP51, both of which lack receptor potentiation activity. Only HsFKBP52 and DrFKBP52-A111V, both of which potentiate receptor activity, retain an open docking conformation compatible for protein–protein interaction. These predictive data suggest that DrFKBP52-A111V, in a manner comparable to HsFKBP52, forms a functionally important contact surface via the FK1 hydrophobic notch with other components within the heterocomplex. Human FKBP51 and DrFKBP52 lack this contact due to the altered loop conformation imposed by occluding leucine and proline, inhibiting adequate protein–protein interaction.

We propose that the HsFKBP52 proline-rich loop, also referred to as the β4–β5 loop, via the open conformation that exposes the hydrophobic notch, forms a specific contact with the receptor ligand-binding domain (LBD). This contact augments the steroid receptor response to hormone. We know that HsFKBP52 binds Hsp90 directly, and through this interaction, selectively potentiates the receptors. Additionally, studies have shown that FKBP52-dependent potentiation is localized in the receptor LBD [5,23]. Since HsFKBP52 selectively potentiates the hormone response in AR, GR, and PR, but not the activity of the estrogen receptor, we conclude that the relevant FK1 interaction for potentiation is receptor-specific, and not exclusively through Hsp90. We presume that the hydrophobic notch that forms above the proline loop region could allow for a more efficient interaction, possibly with the receptor LBD. Through this study, we conclude that the proline residues in the loop are not the sole indicator of functionality, but the overall conformation of the proline-rich loop is critical to maintaining FKBP52 potentiation of the steroid hormone receptor activity.

## 4. Materials and Methods

Yeast strains and β-galactosidase reporter assays. For AR-dependent reporter assays [5], the strains were co-transformed with the indicated plasmids including an AR-dependent β-galactosidase reporter plasmid (pUCΔss-26X), and a high-copy number glyceraldehyde phosphate dehydrogenase (GPD) promoter-regulated yeast expression vector carrying the indicated steroid hormone receptor, HsFKBP51, HsFKBP52, DrFKBP52, and/or site-directed mutants where indicated. Human AR and the mutant AR-P723S were cloned, respectively, into p424GPD and p424TEF [24]. Dihydrotestosterone (DHT) was obtained from Sigma (St. Louis, MO, USA), and dilutions in ethanol were set up to avoid ethanol vehicle concentrations above 1% in the yeast cultures. Hormone-induced reporter activity was measured from yeast extracts as described previously with a single two-hour time point measurement. The data were normalized to cell density by dividing relative light units (RLU) by the optical density (OD) of the cultures at 600 nm (RLU/OD600) and further normalized to percentages. All data shown were generated from at least three biological and technical replicates.

Selection for gain-of-function DrFKBP52 mutants. Error-prone PCR (GeneMorphII; Stratagene, La Jolla, CA, USA) with conditions recommended to produce low-frequency mutagenesis (50–100ng template DNA per reaction) was used to generate the DrFKBP52 mutant libraries using p425GPD-DrFKBP52 as a template. The selection strain YNK435 (*MAT*a: *ura3*-*52, lys2*-*801, ade2*-*101, trp1*-*63, his3*-*200, leu2*-*1, pdr5*::*GT3Z, his3*::*GT3H,* p425TEF-AR-P723S) was then co-transformed with 400 ng of the PCR product and 100 ng of linearized p424GPD expression vector. Gain-of-function mutants were identified by colony growth on synthetic complete medium lacking tryptophan, leucine, and histidine (SC-WLH) supplemented with 10 nM DHT and 10 mM 3-amino-1,2,4-triazole (3-AT). The concentrations of both DHT and 3-AT were chosen based on concentrations that produced the maximum difference in growth between strains expressing either HsFKBP52 or *Dr*FKBP52.The colonies containing DrFKBP52 that grew comparable to those containing HsFKBP52 were selected. The gain-of-function phenotype was then confirmed by assessing the ability to potentiate AR-dependent reporter gene expression in the selection strain. The DrFKBP52 mutant plasmids were then extracted from yeast lysates using standard procedures, and the extracted mutant plasmids were transformed into strain W303a expressing wild type AR and an AR-responsive β-galactosidase reporter plasmid (pUCΔss-26X). Transformants were assayed for potentiation of hormone signaling, as described above. Those mutated DrFKBP52 genes that retained the ability to potentiate AR activity in this clean genetic background were then sequenced. The identified mutations were then put in individually and in combination into the *Dr*FKBP52 gene by site-directed mutagenesis to determine the mutation or combination of mutations that confer the gain-of-function. Mutations were introduced by site-directed mutagenesis (QuikChange II XL; Stratagene, La Jolla, CA, USA) into the HsFKBP52 or DrFKBP52 genes directly in the yeast expression vector (p423GPD).

Receptor-mediated reporter assays in mouse embryonic fibroblasts. Mouse embryonic fibroblasts derived from FKBP52 knockout mice (52KO MEF) [6] were cultured in 5% CO2 in HyClone minimal essential media/Eagles essential salt solution with 2 mM L-glutamine (Thermal Scientific, Logan, UT, USA) supplemented with 10% charcoal/dextran treated fetal bovine serum (FBS) (HyClone, Logan, UT, USA) 24 h prior to the transfection. Cells were cultured in 6-well plates until they were 80% confluent. They were transfected in triplicate using lipofectamine 2000 (Invitrogen, Carlsbad, CA, USA) according to the manufacturer’s protocol. Transfections were performed for 5 h at a DNA to lipofectamine ratio of 1:3 in MEM-EBSS lacking FBS. The transfection cocktail was mixed as follows: 50 ng of the constitutive β-galactosidase reporter plasmid (pCMVβ; Clonetech, Mountain View, CA, USA), 400 ng of the firefly luciferase reporter driven by the androgen-dependent Probasin promoter (pT81; American Type Culture Collection, Manassas, VA, USA), and 800 ng of the pCI-Neo plasmid (Promega, Madison, WI, USA) with or without expression of human AR, HsFKBP52, DrFKBP52, and/or DrFKBP52 mutants. Twenty-four hours after transfection, the medium was replaced with medium containing 10 pM DHT, or a range of doses as indicated. Sixteen hours after hormone addition, cells in each well were lysed using 100 μL mammalian protein extraction reagent (M-PER) (Pierce, Rockford, IL, USA) supplemented with complete ethylenediaminetetraacetic acid (EDTA)-free mini protease inhibitor (Roche, Manheim, Germany) and spun at 15,000 rpm in a 4 °C microcentrifuge to remove impurities. Luciferase expression was quantified by mixing 40 μL cell lysate with 100 μL of luciferase assay reagent (Promega, Madison, WI, USA) in a single well for each sample on a 96-well plate. β-galactosidase expression was quantified by adding 20 μL cell lysate with 100 μL of Gal Screen Reagent (Tropix, Bedford, MA, USA). The 96-well plates were incubated at room temperature, followed by quantification of luminescence by a microplate luminometer (Synergy HTX Multi-mode reader, BioTek, Winooski, VT, USA). Luminescence was measured in relative light units (RLU). The data were normalized for transfection efficiency (luciferase RLU/ β-galactosidase RLU) and further normalized to a percentage. The data shown represent five independent experiments of at least two separate samples, and figures are composite graphs representing data from at least three independent experiments.

Western immunoblots. Western immunoblots were performed by way of standard procedures. Mammalian cells were lysed in M-PER (Pierce, Rockford, IL, USA) supplemented with Complete mini EDTA-free protease inhibitors (Roche, Indianapolis, IN, USA) and protein concentrations were determined by Coomassie Plus Protein Assay (Pierce, Rockford, IL, USA). After separation on 10–20% Criterion gels (Bio-Rad, Hercules, CA, USA) proteins were transferred to polyvinylidene difluoride membranes. The primary antibodies used include mouse monoclonal α-FKBP52 (HI52D), rabbit α-human AR (Santa Cruz Biotechnology, Santa Cruz, CA, USA), and Glyceraldehyde-3-phosphate dehydrogenase (6C5; Biodesign International, Saco, MN, USA). Alkaline phosphatase-conjugated goat anti-mouse or anti-rabbit antibodies were used in conjunction with Immune-Star AP substrate (Bio-Rad, Hercules, CA, USA) before detection on X-ray film.

I-TASSER Homology Modeling. The template and target proteins were selected and extracted from NCBI:Pubmed (http://www.ncbi.nlm.nih.gov/pubmed). The basic local alignment search tool (BLAST) was used to determine template sequence homology by specific parameters. To create the model, a sequence alignment file between the target and selected template sequence (HsFKBP51 and DrFKBP52, respectively, or HsFKBP52 and DrFKBP52, respectively) is required. To this aim, we used another online server, the multiple sequence alignment ClustalW2 (http://www.ebi.ac.uk/Tools/msa/clustalw2/). The sequence for the selected template was obtained directly from the BLAST webpage or from the PDB website. The DrFKBP52 and DrFKBP52-A111V amino acid sequences were submitted to I-TASSER for homology modeling (http://zhanglab.ccmb.med.umich.edu/I-TASSER/). The results were then individually assessed.

## Abbreviations

AR	Androgen receptor
GR	Glucocorticoid receptor
PR	Progesterone receptor
FKBP	FK506-binding protein
Hsp	Heat shock protein
TPR	Tetratricopeptide repeat
BF3	Binding function 3
H1-H3	Helix1-helix3
FK1	FK506-binding domain 1
FK2	FK506-binding domain 2
PCR	Polymerase chain reaction
GPD	Glyceraldehyde phosphate dehydrogenase
DHT	Dihydrotestosterone
OD	Optical density
3-AT	3-amino-1,2,4-triazole
FBS	Fetal bovine serum
MEF	Mouse embryonic fibroblasts
52KO	*Fkbp52* knockout
EDTA	Ethylenediaminetetraacetic acid
RLU	Relative light units
LacZ	β-galactosidase
PPIase	Peptidyl-prolyl cis/trans isomerase
Dr	Danio rerio
Hs	Homo sapiens
SC-WL	Synthetic complete medium lacking tryptophan, leucine and histidine
